# Case of Racemiferous Hydatids of the Uterus

**Published:** 1845-02

**Authors:** J. K. Mitchell


### MEDICAL EXAMINER,

AND

### RECORD OF MEDICAL SCIENCE.

NEW SERIES—No. II.—FEBRUARY, 1845.

---

### ORIGINAL COMMUNICATIONS.

### *Case of Racemiferous Hydatids of the Uterus.* Reported by J. K. Mitchell, M. D.

On the 10th of July I was called to the case of Mrs. T———, who had returned a few days before from a visit to "the South." She complained of nausea, such as usually affects females during utero-gestation, but of greater intensity and prolongation. There was also an unusual degree of tenderness to the touch in the hypogastric region, extending to the right iliac fossa. A careful examination of the part by palpation, presented no unusual conformation, induration, or tumefaction. The history of the case led to the supposition of the existence of a pregnancy of about a month's duration, as, previously to that period, her catamenial regularity and perfect health left no doubt of an unimpregnated condition.

Aperient medicines, to regulate a costive state of the bowels, and antacids, for an acid condition of the stomach, with sinapisms as revellents, relieved the more pressing symptoms. On the 18th of July my attention was called to a small tumour on the right side, about half way from the *symphysis pubis* to the anterior superior spinous process of the *os ilii*, in a right line. It was then about the size of a turkey's egg. The part was painful to the touch, ached when at rest, and suffered from attempts to alter the position in bed. There was a remarkable frequency (120) of the pulse, some heat of surface, and an anxious expression of countenance. The tongue was dry, but clean, the thirst moderate, the nausea irrepressible; and slight mental incoherency, with restless movements of the head and hands, indicated much disturbance of the innervation.

The application of leeches and a poultice relieved in some measure the local suffering, and an antispasmodic prescription abated the restlessness.

No examination exteriorly over the symphysis pubis, by palpation or percussion, could detect any uterine enlargements; so that I was led to suppose that there was an acute affection of the right ovary complicated with peritonitis, and therefore placed the patient entirely at rest, and used such antiphlogistic measures as her feebleness would permit.

On the 22d of July the *uterus* was perceptibly enlarged, occupying a position entirely to the right of the median line, and extending from the place of the tumour first discovered to the *symphysis pubis.*

On the 26th, an examination *per vaginam* was permitted, and resulted in the certainty that the uterus was enlarged, and connected with the tumour, as the movement of the one altered, in a corresponding manner, the position of the other.

On the 28th, it was found that the rapid increase in the size of the *uterus* had obliterated the exterior vestiges of the lesser tumour, and that the former occupied the whole of the right hypogastric region, and rising above the umbilicus, extended a little way to the left of the *linea alba.*

Irritation, and probably pressure suddenly produced, interfered with the power of micturition, and a catheter was used to withdraw the urine, of which the quantity was scanty, and the quality offensive.

As the case had by that time assumed a difficult and threatening shape, I asked for the assistance of my friend Dr. R. M. Huston; and accordingly, on the 30th of July, a consultation was held, and another very careful examination made, exteriorly and *per vaginam.*

The uterus had by this time acquired such a size as to fill nearly the whole abdominal cavity on the right side, while it extended about two inches to the left of the *linea alba*, without any obliquity in the position of the *os tincæ*, to explain the presence of the body of the *uterus* on the right side above.

The history of the case, the short period of time since the cessation of the *menses*, the singular tumour on the right side, and the preternatural rapidity of the developement of the uterus, rendered the *diagnosis* obscure; but on the whole, we were disposed to believe that a dropsy of the right ovary had extended to the uterus, or that there was a rapid production of a mole *in utero.* The absence of any *fremitus* on percussion, and the escape of a little unmixed blood, misled as to hydatids; and the rapidity of developement, and failure to excite motion, left no doubt as to the absence of a *fœtus.*

On the 7th of August contractions of the *uterus*, with the usual pains, announced expulsive efforts, and in the course of the night an immense body of hydatids were expelled. There were many thousands of these vesicles attached to each other, or to a common membrane, so as to appear like bunches of grapes. They varied in size from almost imperceptible globules to the dimensions of large grapes. A few had acquired the volume of a pigeon's egg, while one or two were as large as a hen's egg. They were transparent, uniform, and without nucleoli or apparent organs, and might be properly termed *racemose acephalocysts.*

Hemorrhagy and after pains, as in ordinary cases of labour, followed the expulsion of the hydatids, without causing any abatement of the abdominal tenderness or frequency of pulse.

On the following day signs of puerperal peritonitis became obvious; an addition was therefore made to the consultation by calling in Dr. Joseph Hartshorne, and such measures taken as were possible in the exhausted condition of the patient.

On the 9th the case ended in death, and in thirty-two hours thereafter an autopsy took place, for the following minute of which I am indebted to Dr. Charles Huston, who conducted the dissection.

On opening the cavity of the peritoneum it was found to contain about ten ounces of turbid serum, mixed with pus, of which latter a less diluted portion was found in the pelvic cavity. The right ovary was completely disorganized, nothing having been left of it but the exterior membrane, which was found ruptured, and appeared to have been filled with pus, of which a part still remained. The left ovary was enlarged and softened. It presented, when cut into, a very beautiful, perfectly developed, *corpus luteum.*

The *uterus* was about the size of that organ as it is usually found a day or two after delivery. The interior presented a rough surface at the fundus, as if there had been an attachment of the membrane or of some of the hydatids to it, and that part was partially covered with coagulated blood. The cervix was of an unusually dark hue, but not softer than usual.

This case is interesting for several reasons,—

1*st.* Because it gave no signification of its character by the discharge, from time to time, of single vesicles, or by intermittent gushes of water, produced by their accidental rupture, an event not unusual in such cases.

2*d.* Because it was obviously a consequence of impregnation; a blighted ovum having given origin to the disease, as evinced by the presence of the membranes, to which the vesicles were attached, and by the perfect developement of a *corpus luteum.*

*3d.* Because of the very rapid developement, first of an ovary, then of the uterus.

*4th.* Because of the severe constitutional disturbance, which, as proved by the history of other cases, marks the presence of hydatids *in utero,* and is not commonly found either in uterine dropsy or pregnancy.

*5th.* Because there remained *no traces* of a fœtus, and no vestiges of an ovum, except the transparent membrane to which the vesicles were attached; the most careful examination of which could not, *per se,* have given evidence of an ovarian origin.

